# Measles and rubella: From global health challenges to advancements in molecular diagnostics in the elimination era

**DOI:** 10.1016/j.omtn.2025.102698

**Published:** 2025-09-02

**Authors:** Shivani Sharma, Yuba Raj Pokharel

**Affiliations:** 1 Faculty of Life Sciences and Biotechnology, South Asian University, Rajpur Road, Maidan Garhi, New Delhi 110068, India

**Keywords:** MT: Oligonucleotides: Diagnostics and Biosensors, Measles, Rubella, CRISPR-Cas, Surveillance, RDT, POCT, NAAT

## Abstract

Measles and rubella are highly contagious viral infections with significant public health implications, particularly in low- and middle-income countries. Despite the availability of effective vaccines, these diseases continue to cause periodic outbreaks, contributing to substantial global morbidity, mortality, and economic burden. Immunization programs have drastically abridged disease incidence; however, gaps in vaccination coverage and surveillance systems deter complete elimination. The economic impact of outbreaks includes direct healthcare costs and indirect societal losses, emphasizing the need for robust disease control strategies. Accurate and timely diagnosis is pivotal to measles and rubella elimination efforts. Current diagnostic approaches range from conventional RT-PCR (including multiplex and real-time formats), ELISA, and plaque reduction neutralization test (PRNT), to emerging methods such as isothermal amplification loop-mediated isothermal amplification, recombinase polymerase amplification), CRISPR-Cas systems, next-generation sequencing (NGS), microfluidics, and lateral flow assays. Despite their sensitivity, many of these methods require complex infrastructure and skilled personnel, limiting their utility in field settings. To bridge diagnostic gaps, there is an urgent need for rapid, affordable, and field-deployable nucleic acid-based diagnostics that are simple to use with minimal training. Innovations like CRISPR-Cas and microfluidic platforms hold promise for decentralized testing and real-time surveillance, potentially transforming global measles and rubella elimination programs for the future.

## Graphical abstract

**Figure:**
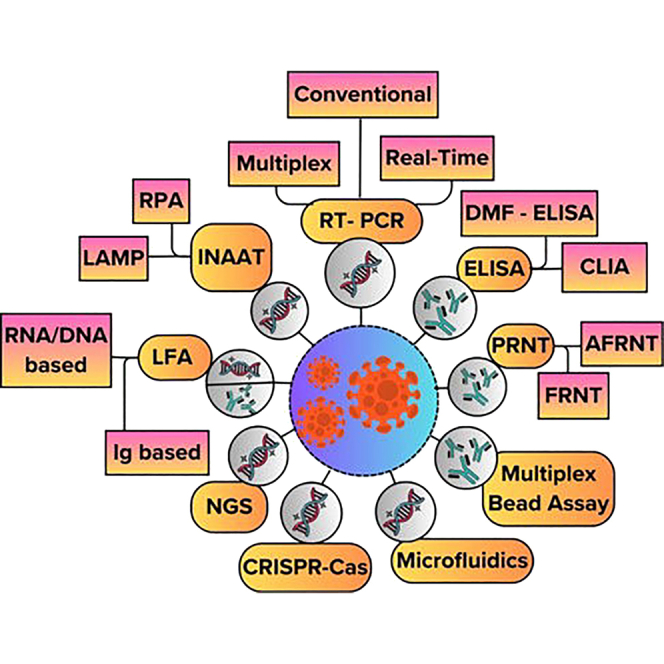

## Teaser

Measles and rubella cases surge globally despite vaccination efforts, with diagnostic limitations hindering elimination goals. This review examines the evolution from conventional serology to cutting-edge molecular technologies, including CRISPR-Cas, digital PCR, and next-generation sequencing. Although emerging platforms offer enhanced sensitivity and point-of-care potential, bridging implementation gaps remains critical for global elimination.

## Introduction

**Figure 1:**
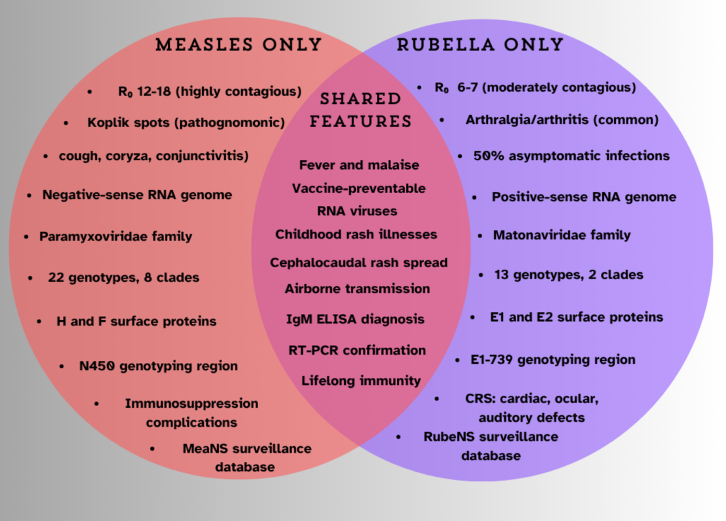
Comparative Venn diagram highlighting distinguishing and shared features of measles and rubella The diagram emphasizes key clinical implications, diagnostic markers, and public health concerns, aiding in differential diagnosis and reinforcing the importance of vaccination and surveillance.

Measles and rubella are highly contagious viral diseases posing significant global public health challenges despite extensive vaccination and control efforts. According to the World Health Organization (WHO) and the United Nations International Children’s Emergency Fund (UNICEF) analysis, 127,350 measles cases were reported in the European Region for 2024, double the number of cases reported for 2023 and the highest number since 1997. Children under 5 years accounted for more than 40% of the reported cases, with 38 deaths reported based on preliminary data received as of March 2025.^1^ Both diseases present distinct viral features, clinical manifestations, and complications ranging from mild to severe. A comparative overview of measles and rubella is illustrated in Figure 1, highlighting their distinguishing and shared clinical, virological, and epidemiological features. Elimination is defined as the absence of endemic transmission for >12 months in a country with adequate surveillance capacity. However, achieving this goal requires robust surveillance systems underpinned by timely, accurate laboratory diagnostics that inform routine immunization efforts, supplementary immunization activities (SIAs), and outbreak responses.^2^ Current diagnostic limitations, particularly in early infection stages and resource-limited settings, continue to hinder effective outbreak control and elimination verification.^3^

The COVID-19 pandemic has accelerated advances in molecular diagnostics and point-of-care testing, creating new opportunities for measles and rubella detection. Emerging technologies such as CRISPR-based assays, isothermal amplification, and next-generation sequencing (NGS) offer potential solutions to long-standing diagnostic challenges. This review examined the progression of diagnostic approaches for measles and rubella, spanning from conventional serological and RT-PCR techniques to recent innovations in rapid diagnostic tests (RDTs) and advanced molecular detection technologies. These methodologies were systematically assessed in chronological order of their development, with performance characteristics, practical applications, and inherent limitations evaluated across diverse clinical and laboratory environments. The analysis underscored the essential function of quality assurance programs, particularly external quality assessment (EQA) schemes, in maintaining diagnostic reliability and accuracy standards. With ongoing measles and rubella threats and renewed elimination commitments under Immunization Agenda 2030 (IA2030), innovative diagnostic solutions are critical for enhancing detection speed, accuracy, and accessibility. This review provides timely insights into the current challenges and emerging diagnostic opportunities to support effective public health responses and advance global elimination goals.

## Measles: Virology, genetic characterization, and clinical features

The measles virus (MeV) is a single-stranded, negative-sense RNA virus with a genome of approximately 15.9 kb belonging to the *Morbillivirus* genus of the Paramyxoviridae family.^4^ It causes a highly contagious acute respiratory illness with significant morbidity and mortality, is human specific with rare transmission to nonhuman primates, and is airborne with no known animal reservoirs.^5^

The MeV genome encodes six proteins: nucleocapsid (N), phosphoprotein (P), matrix (M), fusion (F), hemagglutinin (H), and large polymerase (L). The H protein mediates cellular attachment via CD150 and nectin-4 receptors, whereas the F protein enables membrane fusion.^6^ These surface glycoproteins are primary targets for neutralizing antibodies, contributing to MeV’s antigenic stability as a single serotype. In cell cultures, MeV forms multinucleated giant cells (syncytia) through cell-cell fusion and increases heat shock protein expression while being linked to chromosomal fragmentation.^7^^,^^8^

WHO classifies MeV strains into eight clades (A–H) comprising 22 genotypes.^9^ Genotyping involves sequencing the N450 region or the full-length H gene.^10^^,^^11^ Vaccine strains belong to genotype A. Global measles surveillance shows that only genotypes B3 and D8 have been circulating since 2021. Seven formerly active genotypes (C2, D2, D3, G2, H1, H2, and D4) ceased circulation by 2020.^12^

A single infected individual can transmit the virus to 12–18 others in susceptible populations.^13^^,^^14^ Measles begins with a 2- to 4-day prodromal phase featuring fever and the “three Cs”: cough, coryza, and conjunctivitis.^15^ The characteristic erythematous, maculopapular rash appears 2–4 days after fever onset. Rash begins on the face before spreading to the trunk and limbs, then fading over 3–5 days. Koplik spots appear in 70% of cases, usually 1–2 days before the rash.^16^ Common complications include otitis media (7%–9%), pneumonia (1%–6%), diarrhea (8%), post-infectious encephalitis (1 in 1,000), and subacute sclerosing panencephalitis (1 in 10,000). Death occurs in roughly 1 in 1,000 infections. High-risk populations include infants, adults over 20 years, pregnant women, malnourished children, and immunocompromised individuals, with mortality rates of 1%–15%.^17^ Measles during pregnancy increases the risk of miscarriage, preterm labor, low birth weight, and maternal death.^18^

## Rubella: Virology, genetic characterization, and clinical features

Rubella virus (RuV) is an enveloped, positive-sense, single-stranded RNA virus in the genus *Rubivirus* (family Matonaviridae).^19^ It exists as a single stable serotype, making the 1960s' vaccines still effective today. Humans are the only known natural host, although related viruses have been found in bats and other mammals.^20^ The 9.8kb genome encodes structural proteins, including capsid protein and envelope glycoproteins E1 and E2, which form spikes crucial for cell entry.^9^^,^^21^ The immune response primarily targets the E1 glycoprotein, containing key antigenic determinants.^22^^,^^23^ Specific cellular receptors are largely unknown, although myelin oligodendrocyte glycoprotein may serve as a central nervous system receptor.^24^

Rubella viruses are classified into two clades and 13 genotypes based on 8%–10% sequence variation. Genotyping uses 739 nucleotides within the E1 gene (E1–739) for epidemiological surveillance and elimination verification. Genotypes 1E and 2B are currently the most common worldwide.^25^^,^^26^ Between 2016 and 2018, circulating genotypes declined from five to two due to elimination efforts, although global surveillance remains incomplete in regions like Africa and the Eastern Mediterranean.^27^^,^^28^^,^^29^

The term “rubella” comes from the Latin word *rubellus*, meaning “little red,” and was first introduced in 1866 by Henry Veale.^30^ Rubella is an acute viral illness often misdiagnosed as measles, dengue, parvovirus B19, human herpesvirus (HHV)-6, or scarlet fever. Approximately 50% of infections are asymptomatic.^31^ When present, symptoms typically begin with a 1- to 5-day prodrome, including low-grade fever, headache, malaise, coryza, conjunctivitis, and lymphadenopathy, followed by a mild maculopapular rash that spreads in a cephalocaudal pattern.^32^ Arthralgia or arthritis may also occur, particularly in adolescents and adults.^31^ Although generally mild, primary rubella infection during early pregnancy can lead to miscarriage, stillbirth, or congenital rubella syndrome (CRS), which carries significant medical and public health implications.^33^

## Global burden of measles and rubella

**Figure 2:**
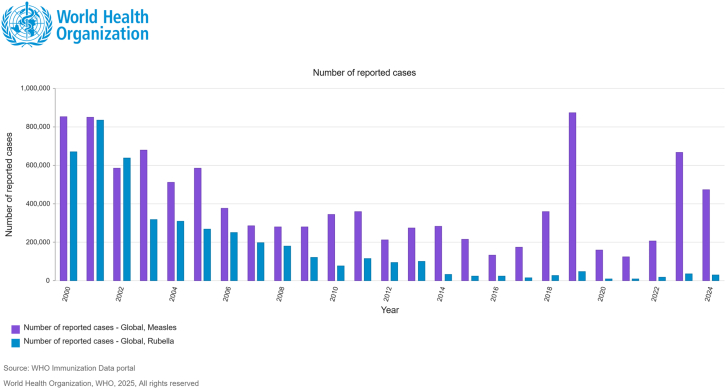
Reported global cases of measles and rubella from 2000 to 2024, compiled from the WHO/UNICEF Joint Reporting Form (JRF) on Immunization These data reflect annual trends used to inform immunization strategies and policy decisions by global health advisory bodies such as the Strategic Advisory Group of Experts on Immunization (SAGE). Source: World Health Organization. WHO Immunization Data Portal. Geneva: WHO; 2025. Available from: https://immunizationdata.who.int/ (accessed July 17, 2025).

**Figure 3:**
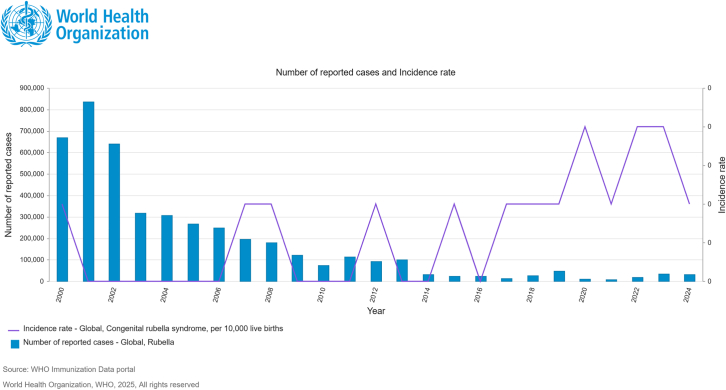
Reported global and regional cases and incidence of CRS, compiled from the WHO/UNICEF Joint Reporting Form (JRF) on Immunization Country-reported data, including historical records, are continuously updated as received and are used to generate annual global and regional trends. These data inform policy recommendations by expert bodies such as the Strategic Advisory Group of Experts on Immunization (SAGE), supporting evidence-based decisions on vaccination strategies, including the consideration of booster doses and target age groups. Source: World Health Organization. WHO Immunization Data Portal. Geneva: WHO; 2025. Available from: https://immunizationdata.who.int/ (accessed July 17, 2025).

Since 2024, all WHO regions have reported increased numbers of measles cases, with 395,521 laboratory-confirmed cases. Also, 16,147 cases were reported during the first 2 months of 2025.^34^^,^^35^^,^^36^^,^^37^ As of July 15, 2025, 40 US jurisdictions reported 1,309 confirmed measles cases with 29 outbreaks. Of these cases, 88% (1,151 of 1,309) were outbreak associated.^36^^,^^38^^,^^39^^,^^40^ Rubella is the leading vaccine-preventable cause of birth defects, accounting for an estimated 100,000 infants born with CRS each year worldwide.^41^ WHO reports a global CRS burden of approximately 100,000 cases annually.^25^ It is estimated that 32,000 to 100,000 cases of CRS occur annually, depending on the modeling methodology utilized.^42^ As shown in Figure 2, reported global cases of measles and rubella from 2000 to 2024 indicate annual trends that inform immunization strategies and policy decisions by the Strategic Advisory Group of Experts on Immunization (WHO/UNICEF, JRF, 2024, https://immunizationdata.who.int/). Figure 3 shows the global and regional trends in reported CRS cases and incidence based on country-reported data to the WHO/UNICEF Joint Reporting Form.

## Health impact of the measles and rubella immunization programs

In 2012, the World Health Assembly approved the Global Vaccine Action Plan, aiming to eliminate measles, rubella, and CRS in at least five of the six WHO regions by 2020.^43^ Although all WHO regions set measles elimination goals, only two had specific targets for rubella.^44^ The COVID-19 pandemic severely disrupted routine vaccination programs and SIAs.^45^^,^^46^^,^^47^^,^^48^ By 2024, measles-containing vaccine first dose (MCV1) global coverage had reached 84%, although this remained below the 95% target needed for herd immunity.^49^ Coverage of measles-containing vaccine second dose (MCV2) also rose steadily to 76% by 2024. During 2000–2023, measles vaccination prevented an estimated 60.3 million deaths globally.^50^ By the end of 2024, 84% of children had received one dose of MCV by their second birthday.^50^^,^^51^^,^^52^^,^^53^ Globally, in 2024, there were 14.3 million children missing out on any vaccination, the so-called zero-dose children.^54^ More than half (over 50%) of the zero-dose children are concentrated in just nine countries: Nigeria, India, Sudan, Democratic Republic of Congo, Ethiopia, Indonesia, Yemen, Afghanistan, and Angola.^53^^,^^55^ Measles vaccination had the most substantial impact on reducing disease burden in China from 1974 to 2024.^56^ Based on the calendar year approach, there was a 95.59% reduction in cases, a 90.18% reduction in deaths, and a 90.05% reduction in measles-related disability-adjusted life years (DALYs). Field evaluations show that MCV1 given at or after 12 months provides a median effectiveness of 93% (range: 39%–100%).^57^ The MCV2 increases protection to 97% median effectiveness (range: 67%–100%).^58^

In line with these findings, the WHO recommends that all countries adopt a two-dose schedule for MCVs.^59^ Despite the high two-dose measles vaccination coverage, immunity gaps exist in adolescents and young adults due to waning vaccine immunity. Vaccine-induced immunity lasts an average of 15.3 years (95% confidence interval [CI]: 10.8–20.2) versus lifelong natural immunity (average: 208 years).^60^ Studies show seropositivity rates as low as 54.3% in adults aged 20–26 years. A third dose at 18–20 years has been proposed to address these immunity gaps in highly vaccinated populations.^61^

Between 1965 and 1967, Stanley Plotkin developed the RA27/3 rubella vaccine, which remains the most widely used rubella vaccine worldwide.^62^ The antibody response rate to a single dose exceeds 95%, while a second dose raises the response rate to nearly 100%.^63^ As of January 2024, 175 of 194 countries had introduced rubella vaccines and the global coverage was estimated at 69%.^25^^,^^32^^,^^41^^,^^42^^,^^53^^,^^64^ Immunity remains detectable for over 21 years despite a decline in rubella virus-specific immunoglobulin G titers.^65^ A modeling study estimated that rubella immunization from 2000 to 2030 could prevent 1.2 million CRS-related deaths (95% CI: 0.47–2.1 million) and avert 86 million DALYs (95% CI: 56–170 million) across 112 countries.^66^ Universal rubella vaccination implementation in the remaining 19 countries is projected to avert nearly 1 million CRS cases by 2055.^67^^,^^68^

**Figure 4:**
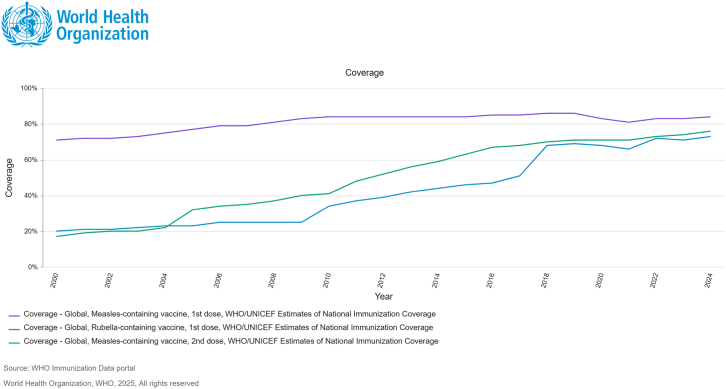
Overview of global immunization coverage for MCV1, MCV2, and rubella vaccine from 2000 to 2024 Data illustrate progress in vaccine uptake worldwide and are used to monitor coverage trends and guide immunization policies. Source: World Health Organization. WHO Immunization Data Portal. Geneva: WHO; 2025. Available from: https://immunizationdata.who.int/ (accessed July 17, 2025).

To strengthen disease control, WHO prioritizes high mumps control. Consequently, 121 countries have incorporated combined measles-mumps-rubella (MMR) vaccines into national immunization programs.^69^^,^^70^ These vaccines simultaneously protect against all three diseases while improving coverage by reducing required injections and healthcare visits.^71^ As depicted in Figure 4, the global immunization coverage for MCV1, MCV2, and RCV from 2000 to 2024 highlights trends in vaccine uptake, which are instrumental in shaping immunization policies and monitoring global coverage. Consequently, the Measles & Rubella Initiative developed the 2021–2030 strategic framework under the broader IA2030. It outlines a global strategy to improve health outcomes through immunization, with the elimination of measles and rubella identified as critical objectives.^72^ Measles, in particular, is positioned as a “tracer” for the performance of immunization programs, as outbreaks often signal gaps in routine childhood immunization coverage.^72^^,^^73^

## Measles and rubella microarray patches: An alternative to syringe/needle system

Traditional measles and rubella vaccines require cold storage (2°C–8°C), reconstitution, and skilled injection personnel, posing challenges in low- and middle-income countries (LMICs).^74^ Measles and rubella microarray patches are coin-sized patches with micro-projections that deliver vaccines painlessly through the skin via thumb pressure.^75^ They are thermostable, needle-free, require no reconstitution, and can be administered by minimally trained staff.^76^ Phase 2 trials showed comparable safety and immunogenicity to injections in children ≥9 months. Key barriers identified include a limited workforce, service gaps, and low demand.^77^ Successful implementation requires feasibility data, policy updates, and programmatic support.^78^^,^^79^^,^^80^

## Cost-effectiveness of the measles and rubella immunization programs

Economic evaluations consistently demonstrate that measles and rubella vaccination programs represent highly cost-effective public health interventions. These programs deliver substantial financial benefits across diverse global settings.^81^^,^^82^^,^^83^^,^^84^^,^^85^^,^^86^ Measles outbreak costs vary significantly by region. Costs range from US$2,979 per case in Brazil to over US$33,000 per case in the United States.^87^^,^^88^ Annual expenditures exceed US$2 million even during low-incidence periods. Cost-effectiveness analyses reveal remarkable return on investment. Global data from 73 LMICs show that US$58 saved per US$1 invested in measles immunization. China’s Expanded Program on Immunization (1974–2024) demonstrated benefit-cost ratios of 27.75 from a societal perspective and 15.44 from a healthcare perspective.^56^ For rubella prevention, economic modeling indicates significant potential savings. Middle-income countries can save US$4,200–57,000 per case annually.^89^^,^^90^ High-income settings show savings up to US$140,000 per lifetime case prevented. Program optimization strategies enhance cost-effectiveness through multiple approaches.^91^^,^^92^^,^^93^ These include geographic information system (GIS)-assisted microplanning using satellite imagery, community engagement through religious leaders, and optimal multi-dose vial utilization.^94^^,^^95^ Integration of routine immunization with financial incentives particularly improves vaccine uptake among economically disadvantaged populations.^96^ At less than US$1 per child, measles vaccination remains one of the most cost-effective public health investments globally.^54^ It delivers exceptional value in preventing morbidity, mortality, and associated healthcare expenditures.

## Global laboratory-based surveillance and diagnostic workflow

Clinical diagnosis of measles becomes increasingly difficult as vaccine coverage increases.^13^ Accurate laboratory diagnosis is essential for effective case management and public health intervention.^97^ The diagnosis of measles can pose significant challenges for healthcare providers, especially for those with limited clinical experience. These difficulties are particularly evident during the early stages of infection, before the characteristic rash appears.^98^ The diagnostic process is further complicated in atypical cases, such as in infants with residual maternal antibodies, individuals who have recently received immunoglobulin, and those who have been recently vaccinated. In immunocompromised patients, the characteristic rash may be absent.^99^ Clinical differentiation is complicated by diseases with overlapping symptoms, including rubella, dengue fever, parvovirus B19, HHV-6, and vaccination reactions. The Centers for Disease Control and Prevention (CDC) defines suspected measles as generalized maculopapular rash, fever ≥38.3°C, and at least one of cough, coryza, or conjunctivitis.^100^ While offering 75–90% sensitivity, its positive predictive value is limited in low-prevalence areas, necessitating laboratory confirmation.^101^ Upon clinical suspicion, immediate infection control measures are implemented, public health authorities are notified, and unique case identifiers are assigned for surveillance tracking purposes.^102^ The CDC recommends that a nasopharyngeal swab, throat swab, or urine specimen, along with a blood specimen, be collected from all patients with clinical features compatible with measles. Nasopharyngeal or throat swabs are preferred over urine specimens.^102^ Detection of measles RNA was most successful when specimens were collected from the first day of rash through 3 days following onset, although real-time RT-PCR remained viable up to 10–14 days after rash onset. A dual testing approach are implemented whereby both serological and molecular methods are employed to maximize diagnostic accuracy.^103^^,^^104^ Laboratory diagnostic approaches can be classified into conventional and advanced methods based on complexity, sensitivity, and application scope discussed further in this review.

Owing to the often asymptomatic or mild rubella infection, surveillance remains challenging and lacks sensitivity.^59^ This is particularly concerning for pregnant women, as infection during the first 16 weeks can cause miscarriage, stillbirth, or CRS.^41^ The wide range of possible congenital abnormality causes makes CRS surveillance difficult, requiring laboratory confirmation. Surveillance quality varies significantly across countries. Of 194 WHO member countries, 122 have met minimum rubella surveillance standards, while only 95 have achieved CRS standards.^64^ Rubella surveillance is typically integrated with measles surveillance due to similar clinical presentations.^44^ CRS surveillance relies primarily on sentinel site-based reporting. Owing to these limitations, rubella and CRS cases are vastly underreported. By 2022, there were 17,407 rubella cases and 1,527 CRS cases officially reported to WHO, showing significant changes from earlier years.^25^ The 2023 measles outbreak in Nigeria highlights vulnerabilities: only 60.6% of the reported cases had a blood specimen collected and just 35.2% reached laboratories within 3 days, far below the 80% timeliness target.^105^

Laboratory processing was coordinated through the Global Measles and Rubella Laboratory Network (GMRLN), which had developed a detailed laboratory manual containing chapters on laboratory testing and regularly updated protocols for standard tests, with the latest version published in 2018.^4^ GMRLN, comprising over 700 laboratories across 191 countries, categorized laboratories into four tiers: sub-national laboratories, national laboratories, regional reference laboratories, and global specialized laboratories (Table 1).^106^ Quality assurance was maintained through a molecular external quality assurance (mEQA) program initiated by the US CDC in 2014 to evaluate the performance of laboratories performing nucleic acid-based methods with annual proficiency testing demonstrating that 97% of the laboratories submitting measles results and 98% submitting rubella results obtained passing scores in 2023.^3^^,^^4^ Case classification is performed based on laboratory confirmation, with confirmed cases requiring positive IgM and/or RT-PCR results or epidemiological linkage to laboratory-confirmed cases, whereas probable cases should meet clinical definitions without laboratory confirmation and should not be epidemiologically linked.^107^ Discarded cases are those with laboratory evidence against measles/rubella or confirmed alternative diagnoses.^108^ RT-PCR genotyping is employed to distinguish between vaccine reactions and infections with wild-type virus, which is critical for determining infectiousness and implementing appropriate control measures. Enhanced surveillance and response protocols are activated for confirmed cases, including contact tracing, exposure assessment, implementation of control measures, and reporting to national and international surveillance systems. Viral genotyping data are submitted to the Measles Nucleotide Surveillance (MeaNS) database (http://www.who-measles.org/) and the Rubella Nucleotide Surveillance (RubeNS) database (http://www.who-rubella.org/) for global monitoring and integration with epidemiological data for outbreak investigation purposes.^107^^,^^109^

**Table 1:** Classification and roles of laboratories in the measles and rubella surveillance network

Serial No.	Laboratory type	Scope	Primary role	Key functions
1	**SNL**	Local or provincial level	Initial testing and screening	Routine specimen testing, preliminary diagnosis, reports to NLs
2	**NL**	Country wide	Confirmatory testing and national surveillance coordination	Confirm SNL results, perform genotyping (in some cases), oversee SNLs, report to RRLs
3	**RRL**	Multiple countries (regional)	Technical and quality assurance support for national networks	Advanced testing, genotyping, quality control, training, data consolidation
4	**GSL**	Global	Highest level of technical expertise and global surveillance support	Molecular characterization, standard development, outbreak response, collaboration with WHO HQ

GSL, global specialized laboratory; HQ, headquarters; NL, national laboratory; RRL, regional reference laboratory; SNL, sub-national laboratory.

The table summarizes the four-tiered laboratory structure established by the WHO Global Measles and Rubella Laboratory Network. Each tier, SNLs, NLs, RRLs, and GSLs, has distinct responsibilities based on scope, technical capacity, and coordination functions to ensure effective surveillance, diagnosis, and outbreak response.

Widely used diagnostic tools like serological assays and real-time PCR have limitations, particularly in early-stage infections, atypical cases, and low-incidence settings.^40^^,^^110^ Inadequate healthcare worker training, delayed specimen collection, and limited molecular diagnostic access compromise surveillance reliability. Data are also influenced by changes in testing practices, reporting policies, or new technologies, affecting longitudinal comparisons. Despite these challenges, surveillance systems remain essential for measles and rubella control programs. Strengthening their sensitivity, coverage, and integration with molecular diagnostic platforms is critical for reliable, timely, and actionable data.

## Clinical specimen types for measles and rubella diagnosis

For measles, clinical samples include throat/nasal swabs, nasopharyngeal aspirates, oral fluids, urine, or peripheral blood mononuclear cells (PBMCs).^103^ When cold chain maintenance is challenging, dried blood spots (DBSs) offer a viable alternative to plasma for IgG-based serological testing.^111^^,^^112^ DBS demonstrated high sensitivity (100% for mumps and measles, 82.5% for rubella) and specificity (100% for mumps and rubella, 87.5% for measles), with strong correlation to plasma results (r = 0.914–0.953). Stability remained high at 4°C (r = 0.889–0.925) and −20°C (r = 0.878–0.951) but decreased at room temperature (r = 0.762–0.872), especially for measles IgG. Hematocrit had no effect on results.^113^ Study limitations include small sample size, limited 120-day storage evaluation, decreased room temperature stability, use of only one testing platform, exclusive focus on IgG detection, and potential pre-analytical variability in field settings. Specimens may also be spotted onto FTA (Flinders Technology Associates) elute micro cards, which preserve viral RNA and inactivate the virus, enabling safe ambient-temperature shipment. MeV RNA can be detected in serum for up to 7 days following rash onset, and in other specimens for up to 2 weeks or more.^114^ Early collection, preferably shortly after rash onset, is strongly advised. Rubella virus detection uses the same clinical specimens as measles, allowing simultaneous investigation of both infections.^104^^,^^115^ The timing of specimen collection relative to vaccination critically affects diagnostic interpretation. Vaccine virus RNA can be detected in respiratory specimens for 7–14 days post-vaccination, whereas vaccine-induced antibody responses may persist for weeks to months.^114^^,^^115^ WHO recommends avoiding serological testing within 6 weeks of MMR vaccination unless RT-PCR confirmation is available.

## Traditional diagnostic methods with limited clinical utility

Although less sensitive and time consuming, virus isolation in cell cultures remains valuable when high viral titers or comprehensive viral characterization are needed. MeV can be cultured from nasal/throat swabs, nasopharyngeal aspirates, urine, or PBMCs using B95a and Vero/hSLAM cell lines.^116^ Ihara et al. (1995) demonstrated that B95a cells enabled successful MeV isolation from 91% of urine samples (10 of 11) when collected within 2 days following rash onset.^117^ Isolation rates declined to 67% for samples collected between days 3 and 5, with no successful isolations beyond day 5. Therefore, collecting high-quality specimens within 5 days of rash appearance is critical for virus isolation efficiency. WHO recommends Vero/hSLAM cells for the global laboratory network as they are not persistently infected with Epstein-Barr virus (EBV) and also support rubella virus propagation.^118^ Vero/hSLAM cells are Vero cells (cell line derived from African green monkey kidney cells) that have been stably transfected with a plasmid encoding the gene for the hSLAM (human signaling lymphocytic activation molecule, also known as CD150). hSLAM is a receptor for both wild-type and laboratory-adapted strains of measles.^119^

For rubella, the virus can be propagated in RK13, SIRC, baby hamster kidney, and Vero or Vero/hSLAM cell lines. Unlike measles, wild-type rubella strains rarely cause visible cytopathic effects, and even adapted strains may take 5 days to form plaques at 35°C.^120^^,^^121^^,^^122^ Therefore, confirmation often requires additional techniques such as RT-PCR, immunofluorescence, or immunocolorimetric assays (ICAs). The latter can produce visible foci within 3 days and match immunofluorescence in sensitivity.^123^ Owing to low rubella virus concentrations in clinical samples and large target regions required for genotyping, virus isolation may be necessary for obtaining sequencing data. However, these cells lack the necessary receptors for most wild-type viruses, accounting for low isolation success rates.^124^^,^^125^

## Diagnostic gold standards and the gaps

The diagnostic methods discussed in this review can be categorized into three fundamental principles: antibody-based detection, neutralization assays, and nucleic acid detection. Although we present these methods chronologically to illustrate technological evolution (Table 2) and how each advance addressed previous limitations, readers should note that methods sharing the same underlying principle often have similar constraints and capabilities.

**Table 2:** Chronological and comprehensive comparison of MR diagnostic methods with commercial kits, performance metrics, and cost-effectiveness analysis

Serial No.	Method	Year	Target	Commercial kits/manufacturers	Sensitivity (%)	Specificity (%)	PPV/NPV (%)^a^^,^^b^	LOD	Sample volume	Sample types	Cost per test (US$)^c^	Equipment cost (US$)	Time to result	Technical requirements	Pros	Cons
1	HI^126^	1940s	Antibodies	Laboratory protocols; no commercial kits	Moderate (60–75)	Moderate (75–85)	Variable by prevalence	N/A	50–100 μL	Serum	5–8	2,000–5,000	1–2 days	Low; basic serology training	Simple setup, historical importance	Outdated, poor sensitivity/specificity
2	PRNT^127^	1960s	Neutralizing antibodies	Laboratory protocols; no commercial kits	Very high (98–100)	Very high (99–100)	M: 98–100/99–100; R: 98–100/99–100	Variable	50–100 μL	Serum	50–80	15,000–40,000	3–5 days	Very high; specialized virology expertise	Gold standard for immunity; WHO reference	Labor intensive, requires BSL-2+, slow
3	ELISA (EIA)^128^^,^^129^^,^^130^	1971	IgM/IgG antibodies	Siemens Enzygnost^d^ (discontinued 2020); Euroimmun; IBL^129^ International; SERION ELISA classic; NovaTec NovaLisa	M: 75–98; R: 78–99	M: 87–99; R: 52–100	M: 75–97/88–99; R: 0.2–1.4/94–99	N/A	10–100 μL	Serum, plasma	15–25	25,000–50,000	2–4 h	Moderate; trained laboratory technician	Widely available, WHO network standard	Lower PPV in elimination settings
4	CLIA^131^	1990s	IgM/IgG antibodies	DiaSorin LIAISON XL; Beckman Coulter Access; Abbott ARCHITECT; Siemens ADVIA Centaur	M: 94–97; R: 90–96^129^	M: 95–98; R: 93–98	M: 95–98/96–99; R: 94–97/95–98	N/A	25–50 μL	Serum, plasma	18–30	80,000–150,000	30–60 min	Moderate; automated platform training	High throughput, automated	Expensive equipment, platform specific
5	ICA	1980s	IgM/IgG antibodies	Research protocols; limited commercial availability	High (85–95)	High (90–98)	Variable by prevalence	N/A	50–100 μL	Serum	8–15	5,000–15,000	2–3 h	Moderate; immunology training	Visual detection, cost effective	Less standardized than ELISA
6	RT-PCR^132^^,^^133^	1990s	Viral RNA	Applied Biosystems TaqMan; Bio-Rad CFX; Roche LightCycler; QIAGEN Rotor-Gene; in-house protocols	M: 94–99; R: 98–100	M: 99–100; R: 100	M: 98–100/97–99; R: 99–100/98–100	10–1,000 copies/mL^e^	140–200 μL	Throat/NP swab, urine, serum	20–40	40,000–120,000	3–6 h	High; molecular biology expertise	Highly sensitive and specific, genotyping capable	Complex, expensive, requires skilled staff
7	FRNT^134^	1995	Neutralizing antibodies	Research protocols; immunocolorimetric methods	Very high (95–100)	Very high (98–100)	M: 98–100/98–100; R: 98–100/98–100	Variable	25–50 μL	Serum	30–60	20,000–50,000	2–3 days	Very high; cell culture expertise	More sensitive than PRNT, vaccine studies	Complex, requires cell culture facilities
8	AFRNT^135^	2005	Neutralizing antibodies	96-Well protocols; limited commercial systems	Very high (95–100)	Very high (98–100)	M: 98–100/98–100; R: 98–100/98–100	Variable	25–50 μL	Serum	25–50	30,000–80,000	2–3 days	High; automated systems	Reduced manual error, higher throughput	Expensive setup, specialized equipment
9	RT-LAMP^136^^,^^137^^,^^138^	2008	Viral RNA	Laboratory protocols; no commercial kits	M: 91–100; R: 95–100	M: 95–100; R: 95–100	M: 95–99/96–100; R: 88–98/95–100	M:30–50 copies/mL; R:380 copies/reaction	2–25 μL	Throat swab, urine, serum	8–15	5,000–15,000	30–60 min	Moderate; isothermal amplification training	Equipment-simple, rapid, cost effective	Contamination risk, primer design complexity
10	RT-RPA^137^	2012	Viral RNA	Laboratory protocols; no commercial kits	M: 94–100; R: 85–95	M: 95–100; R: 90–100	M: 96–99/97–100; R: 88–96/95–99	10–31 copies/reaction	1–5 μL	Multiple specimen types	12–25	2,000–8,000	15–30 min	Moderate; molecular training	Very rapid, high sensitivity	Higher reagent costs, proprietary
11	DMF-ELISA^139^	2016	IgM/IgG antibodies	MR Box (research prototype); custom platforms	M: 81–88; R: 81–88	M: 85–95; R: 85–95	M: 80–90/90–95; R: 80–90/90–95	N/A	<5 μL	Serum, oral fluid	10–20	1,000–5,000	30–60 min	Low; minimal training	Portable, minimal sample volume	Research stage, limited validation
12	Lateral flow RDT^140^^,^^141^	2020	IgM antibodies	Prototype developments (WHO/Gavi evaluation); no commercial products yet	M: 90–95^f^; R: Under development	M: 94–96^f^; R: N/A	M: 85–95/96–99^f^; R: N/A	N/A	5–10 μL	Capillary blood, serum, oral fluid	2–4	0	<30 min	Low; point-of-care use	Field deployable, immediate results	Limited validation, lower accuracy
13	MBA^142^	2021	Multiple antibodies	Luminex MAGPIX; Bio-Rad Bio-Plex; custom panels	M: 90–98; R: 92–99	M: 93–99; R: 95–100	M: 94–98/95–99; R: 96–99/97–100	N/A	1–5 μL	Serum, plasma	20–35	30,000–80,000	2–4 h	High; specialized platform	High throughput, multiplexing capability	Expensive platform, complex data analysis
14	Microfluidic two-stage amplification^143^	2021	Viral RNA	Research prototypes; custom chip fabrication	M: 100^g^; R: Under development	M: 100^g^; R: N/A	M: 100/100^g^; R: N/A	∼10 copies	2.1 μL (RPA) + 10.6 μL (LAMP)	NP swabs, saliva	20–35	10,000–30,000	<60 min	High; microfluidics expertise	Ultra-sensitive, integrated workflow	Complex fabrication, research stage
15	NGS amplicon sequencing^144^^,^^145^	2021	Viral genome	Illumina MiSeq/NextSeq; Oxford Nanopore; custom primer panels	M: 90–100; R: 85–100	M: 95–100; R: 90–100	M: 95–100/96–100; R: 90–100/95–100	Variable (Ct < 30 preferred)	50–200 μL	Multiple specimen types	80–150	50,000–250,000	1–3 days	Very high; bioinformatics required	Genotype specific, established protocols	Labor intensive, limited by primer design
16	NGS probe enrichment^145^	2022	Viral genome	Twist Bioscience; IDT xGen; custom probe panels	M: 95–100; R: 90–100	M: 98–100; R: 95–100	M: 98–100/98–100; R: 95–100/97–100	Variable (viral load dependent)	50–200 μL	Clinical specimens	75 (includes $7 enrichment)	50,000–250,000	2–3 days	Very high; NGS and bioinformatics	Genotype independent, cost-efficient	Probe maintenance, degraded sample failure
17	RT-ddPCR^146^^,^^147^	2023	Viral RNA	Research protocols; no commercial kits	M: 95–100; R: 90–100	M: 98–100; R: 95–100	M: 98–100 R: 95–100	M: 260; R: 460 copies/mL	10–50 μL	Throat/NP swab, urine, wastewater, serum	35–60	60,000–200,000	4–6 h	High; molecular biology and dPCR expertise	Absolute quantification without standards, strain differentiation, precision, multiplexing	Expensive equipment, no commercial kits, limited validation
18	CRISPR-Cas12a detection^148^	2024	Viral RNA	Research protocols; emerging commercial development	M: 96^h^; R: Under development	M: 100^h^; R: N/A	M: 98–100/97–99^h^; R: N/A	31 copies/reaction	1–5 μL	Throat swab, saliva	15–30	5,000–20,000	30–60 min	Moderate; CRISPR training	Ultra-specific, rapid, portable	Limited clinical validation, early development
19	Oxford nanopore sequencing^149^	2025	Viral genome	Oxford Nanopore MinION/GridION; custom library preparation	M: 95–100 (high viral load); R: Under development	M: 95–100; R: N/A	M: 95–100/96–100; R: N/A	Optimal >100 copies/μL	50–200 μL	Clinical specimens	60–120	10,000–50,000	6–24 h	High; real-time sequencing	Portable sequencing, real-time results	Error prone at low viral loads
20	Shotgun metagenomics^145^^,^^149^	2025	Total pathogen DNA/RNA	Illumina platforms; Oxford Nanopore; Ion Torrent	Moderate (70–85)	High (90–95)	Variable (pathogen dependent)	Variable	50–200 μL	Clinical specimens	120–200	50,000–250,000	2–5 days	Very high; advanced bioinformatics	Pathogen agnostic, discovers unknowns	Low efficiency, expensive, complex analysis

This table outlines key assays used for MR detection, including their diagnostic targets, commercial manufacturers, detailed performance characteristics (sensitivity, specificity, PPV/NPV), sample volume requirements, cost per test, turnaround time, technical requirements, advantages, and limitations, along with the year of introduction or significant application. The comparison reflects the progression from classical serology (1940s) to advanced molecular and point-of-care technologies (2025), incorporating recent systematic reviews, manufacturer specifications, and WHO guidelines to support evidence-based diagnostic platform selection.

AFRNT, automatable FRNT; ; BSL, biosafety level; HI, hemagglutination inhibition; LOD, limit of detection; M, measles; MR, measles and rubella; NP, nasopharyngeal; NPV, negative predictive value; PPV, positive predictive value; PRNT, plaque reduction neutralization test; CLIA, chemiluminescence immunoassay; R, rubella.

a PPV/NPV values are highly dependent on disease prevalence and vary significantly between elimination and endemic settings.

b PPV typically lower in elimination settings; NPV higher in endemic settings.

c Costs vary by region, volume, and healthcare setting; ranges reflect global estimates.^81^^,^^252^

d WHO-endorsed until discontinuation; replacement evaluation ongoing.

e Detection limit varies with specimen type and collection timing.

f Based on limited field studies; broader validation ongoing.

g Limited validation study (*n* = 40); requires larger clinical trials.

h Single study validation (*n* = 56); broader clinical validation needed.

## Enzyme immunoassay

Enzyme immunoassay (EIA), also referred to as ELISA, represents a fundamental immunoanalytical technique that exploits the catalytic properties of enzymes to detect and quantify immunologic reactions. In this heterogeneous immunoassay format, one of the reaction components is either non-specifically adsorbed or covalently bound to the surface of a solid phase. The solid phase typically consists of 96-well or 384-well polystyrene microplates. These microplates passively bind proteins through hydrophobic interactions. Commercial ELISA platforms utilize recombinant measles nucleocapsid (N) protein (specifically amino acids 1–525 covering the full-length protein) and rubella E1 envelope glycoprotein (amino acids 1–285) as primary capture antigens. These platforms employ both indirect and capture ELISA formats, with capture assays demonstrating superior specificity by eliminating interference from rheumatoid factor and other non-specific antibodies. The capture format uses anti-human IgM heavy chain antibodies immobilized on a solid phase to specifically capture IgM antibodies, followed by antigen binding and detection using enzyme-conjugated anti-antigen antibodies.^150^ The N protein is selected for its high abundance during acute infection and contains multiple immunodominant epitopes recognized by early IgM responses. For rubella, the E1 glycoprotein’s antigenic domains I and II (amino acids 27–46 and 52–68) are critical for IgM binding specificity.^51^^,^^129^ The recombinant N protein expression typically utilizes bacterial systems (*Escherischia coli*) or mammalian cell lines to maintain proper protein folding and epitope presentation. Post-translational modifications, particularly phosphorylation at specific serine and threonine residues within the N protein, can influence antigen-antibody binding kinetics and assay sensitivity.^151^^,^^152^ For rubella E1 protein, glycosylation patterns are crucial for maintaining conformational epitopes, necessitating eukaryotic expression systems for optimal antigen production.^15^^,^^153^ Measles-specific IgM antibodies are detected with 83%–89% sensitivity and 95%–99% specificity.^130^ Cross-sectional studies demonstrate that sensitivity varies by specimen collection timing, with optimal detection occurring 3–7 days post-rash onset when IgM levels peak.^154^^,^^155^ The analytical sensitivity of modern capture EIAs approaches 0.1–0.5 IU/mL for measles IgM while maintaining low cross-reactivity with other paramyxoviruses. Automated platforms such as the Siemens ADVIA Centaur and Abbott Architect systems provide high-throughput processing with chemiluminescent detection, achieving results within 30–60 min^130^ However, IgM may be absent in up to 25% of cases within 72 h after rash onset, and false-positives can occur in low-incidence areas.^156^ False-positive IgM results are particularly problematic in elimination settings where the positive predictive value decreases dramatically.^155^ Common causes include heterotypic immune responses to other viral infections (parvovirus B19, EBV, cytomegalovirus), autoimmune conditions producing rheumatoid factor, and recent vaccination with live-attenuated vaccines. Some commercial assays demonstrate false-positive rates of 5%–15% when used in low-prevalence populations, necessitating confirmatory testing strategies.^157^ IgG detection confirms recent infection through a 4-fold titer rise or seroconversion in paired sera. IgG quantification typically employs standardized international units (IU/mL) referenced against WHO standards, with protective immunity generally considered at levels ≥200 mIU/mL for measles and ≥15 IU/mL for rubella.^158^ Automated platforms utilize competitive or sandwich immunoassay formats with coefficient of variation <10% for intra-assay precision. For paired sera analysis, specimens should be tested simultaneously using the same lot of reagents to ensure accurate titer comparison.^159^ IgG avidity testing distinguishes recent (low avidity) from past infection or vaccination (high avidity).^160^ IgG avidity testing employs chaotropic agents such as urea or diethylamine to disrupt weak antigen-antibody bonds characteristic of recent immune responses. The avidity index is calculated as the ratio of antibody binding in the presence versus absence of chaotropic treatment, expressed as a percentage.^161^ Low avidity (<30%–40%) suggests primary infection within the previous 2–3 months, whereas high avidity (>60%–70%) indicates past infection or vaccination occurring >4 months prior. Intermediate values (30%–60%) require clinical correlation and may necessitate repeat testing after 2–4 weeks.^162^ Critical quality parameters include appropriate positive and negative controls, calibration against international standards, and documentation of lot-to-lot reagent variation. Laboratories must maintain certification through annual - EQAs and demonstrate competency in both routine and outbreak investigation scenarios.^107^

## Plaque reduction neutralization

The plaque reduction neutralization test (PRNT) is the gold standard for quantitatively assessing measles immunity, offering greater sensitivity than hemagglutination inhibition (HI) assays.^163^ The PRN assay measures the ability of antibodies to neutralize live MeV infectivity in cell culture, typically using Vero cells. Serial dilutions of patient serum are incubated with a standardized viral inoculum, and neutralization is quantified by comparing plaque counts in test wells versus virus-only controls. The assay requires 5–7 days for completion and demands strict biosafety level 2 containment due to live virus handling.^120^ Neutralizing antibody titers ≥120 mIU/mL are generally considered protective, corresponding to approximately 50% plaque reduction.^123^ Limitations include requiring two serum samples weeks apart, reliance on fresh monkey erythrocytes for HI testing, lengthy pre-treatment procedures, and PRN being highly specialized and unavailable in most routine surveillance laboratories.^126^

For rubella, the immunocolorimetric neutralization assay is the gold standard for evaluating neutralizing immunity.^120^ The immunocolorimetric neutralization assay employs cell culture with color-change indicators to detect cytopathic effects, providing a semi-quantitative assessment of neutralizing antibodies. This approach uses metabolic indicators such as neutral red uptake to determine cell viability, eliminating the need for plaque counting while maintaining correlation with classical neutralization methods. The assay can detect as few as 10 plaque-forming units of rubella virus and has demonstrated sensitivity and specificity comparable to immunofluorescent assays.^164^ Postnatal rubella is diagnosed using RT-PCR or virus isolation from nasal, throat, or urine specimens, ideally within 3 days of rash onset. CRS should be suspected in infants with cataracts, congenital heart defects, or hearing loss. CRS diagnosis involves detecting rubella-specific IgM within 6 months or rising IgG titer prior to vaccination. IgM antibodies peak around 5 days post-rash and decline within 8 weeks, whereas IgG persists for life. Maternal IgG wanes by 9 months.^165^^,^^166^

IgM detection is most common, but false-positives occur due to cross-reactivity with parvovirus B19, rheumatoid factor, or heterophile antibodies.^167^ A French study of 5,398 serum samples from 4,104 pregnant women found positive predictive values of only 0.2% (95% CI: 0.0%–0.5%) for IgG seroconversion and 1.4% (95% CI: 0.99%–1.81%) for IgM in primary rubella infection.^31^ IgG avidity testing may distinguish recent from past infection but has limited utility in low-incidence settings.^168^ Also, recent MMR vaccination can produce false-positive IgM results for up to 6–8 weeks post-vaccination due to vaccine-induced antibody responses.^115^^,^^169^ Vaccine-induced IgM typically appears 7–14 days after vaccination and may persist for 4–6 weeks, overlapping with the time frame when wild-type infection would be investigated.^129^ This creates diagnostic challenges, particularly in outbreak settings where recently vaccinated individuals may present with fever and rash due to other causes. IgG avidity testing helps distinguish recent vaccination from natural infection, as vaccine-induced antibodies typically show high avidity within 2–3 weeks, whereas natural infection antibodies mature more slowly.^168^ However, this approach has limited utility in individuals with prior vaccination history or in low-incidence settings where positive predictive values are reduced.

## RT-PCR

RT-PCR combines reverse transcription of viral RNA into cDNA followed by exponential amplification through thermal cycling. For measles and rubella diagnosis, one-step RT-qPCR protocols are preferred, utilizing reverse transcriptase and thermostable DNA polymerase in a single reaction tube with real-time fluorescent detection. This approach minimizes hands-on time, reduces contamination risk, and provides quantitative viral load assessment through cycle threshold (Ct) values.^170^ Standard measles RT-qPCR employs a dual-probe TaqMan chemistry targeting the highly conserved nucleocapsid (N) gene region (nucleotides 1,233–1,462). The forward primer (5′-GCCATGGGAGTAGGAGTGGT-3′) and reverse primer (5′-CTCAGTCCCTCAATCCAATC-3′) flank a 230-base pair amplicon, with the fluorescent probe (5′-FAM-AGCATCTGCAAGCTCCACTCTGCC-TAMRA-3′) providing real-time detection. This region shows <2% sequence variation across all circulating genotypes, ensuring broad reactivity while maintaining an analytical sensitivity of 10–100 copies/mL.^171^ The limit of detection varies by platform and extraction method, with automated systems achieving 95% detection probability at 50–100 copies/mL. Analytical specificity exceeds 99.5% when tested against related paramyxoviruses, including parainfluenza viruses 1–4, respiratory syncytial virus, and human metapneumovirus. The dynamic range spans 6–7 log10 copies/mL, enabling accurate quantification across the full spectrum of clinical viral loads. Quality control measures include extraction controls, amplification controls, and inhibition controls to ensure assay validity.^172^

Real-time RT-PCR, particularly TaqMan-based assays, is the preferred method due to its speed (1–2 h for results), minimal manipulation that reduces contamination risk, and ability to amplify short genomic fragments despite RNA degradation.^173^ Real-time PCR assays incorporating vaccine-specific probes can definitively identify genotype A strains within 2–3 h, crucial for elimination settings where rapid differentiation is essential for outbreak investigation.^132^^,^^174^ The probes utilize SNPs unique to the Edmonston lineage vaccines. Key discriminatory sites include nucleotide positions 1,174 (A→G), 1,200 (T→C), and 1,215 (G→A) within the N gene. Recent reports demonstrate vaccine virus RNA detection up to 448 days post-vaccination, necessitating careful clinical correlation.^132^ The assay demonstrates high sensitivity and specificity (94% and 99%, respectively) for measles.^132^ Multiplex RT-PCR formats prove advantageous in elimination settings where suspected cases may be due to other pathogens.^111^^,^^175^ Multiplex panels targeting measles, rubella, parvovirus B19, HHV-6, and enterovirus provide comprehensive differential diagnosis for febrile rash illnesses.^176^ These assays reduce time to result from days to hours while conserving specimen volume. Syndromic panels demonstrate particular value in elimination settings where clinical diagnosis is unreliable and multiple etiologies must be considered. However, cross-reactivity and reduced individual assay sensitivity require careful validation and interpretation.^111^

For rubella, TaqMan-based and nested RT-PCR assays are highly sensitive for suboptimal samples or low viral loads but require meticulous contamination control.^104^^,^^177^ The E1 gene target (nucleotides 8,731–9,469) utilizes primer sets designed to detect both phylogenetic clades. The forward primer (5′-CTGCGTGACATAAAGGACAAG-3′) and reverse primer (5′-GGTCTCGCACCAAATTGTAG-3′) amplify a 185-base pair region spanning a highly conserved domain within the E1 glycoprotein coding sequence. The probe sequence (5′-VIC-CTCCGTCAATCGTAGGCTCTGTGG-NFQ-3′) targets nucleotides essential for viral envelope function, minimizing false-negative results due to genetic drift.^111^ Rubella RT-PCR faces unique challenges due to lower viremia levels and shorter detection windows compared with measles. The E1 gene target region encodes critical neutralizing epitopes, making it highly conserved across genotypes 1E and 2B.^161^ Alternative targets include the non-structural protein genes and 5′ UTR, which may provide enhanced sensitivity for clinical specimens with low viral loads. Nested PCR approaches can achieve 10- to 100-fold sensitivity improvement but require stringent contamination control protocols.^177^^,^^178^

RT-PCR accuracy is influenced by collection timing, sample type, storage, and transport conditions. False-negative results may occur if samples are collected late, stored improperly, or contain minimal or mutated viral RNA.^174^ RT-qPCR primer design addresses several technical challenges. For measles, the high GC content (52%) of the N gene requires careful primer placement to avoid secondary structure formation. Primers are positioned to span exon-exon junctions where possible. Although the MeV lacks introns, this principle applies to avoiding highly structured RNA regions. The TaqMan probe incorporates a minor groove binder to increase the melting temperature and specificity. Rubella primer design must accommodate the lower viral loads typical of rubella infections (10^2^–10^4^ versus 10^4^–10^6^ copies/mL for measles). The E1 target region was selected for its high expression during the viremic phase and relative stability across genotypes. Alternative primer sets targeting the 5′ UTR are employed for specimens with suspected low viral loads, offering 2- to 5-fold improved analytical sensitivity at the cost of reduced genotyping capability. WHO recommends IgM serology as the primary diagnostic tool, with RT-PCR as complementary. However, RT-PCR can be more reliable during the initial days following rash onset than serology.^179^ Positive RT-PCR results are definitive, whereas negative results require cautious interpretation.

## Advances in measles and rubella molecular diagnostics

The GMRLN established a working group to develop and deploy RDTs for measles and rubella. The group created a framework for introducing RDTs to national surveillance programs.^180^ These tests were expected to improve surveillance timeliness and sensitivity. The network ensured that laboratories could use real-time RT-PCR for case confirmation algorithms. It also enabled the generation of high-quality sequence data to monitor viral transmission pathways. NGS was implemented for enhanced outbreak investigations.^181^ Alternative specimen types, such as DBSs and oral fluid, were validated for use when cold chain maintenance was challenging, and point-of-care testing was developed for decentralized diagnosis in resource-limited settings. This comprehensive workflow provided systematic, quality-assured diagnosis while supporting global elimination efforts. It integrated standardized surveillance and rapid response mechanisms with traditional serological methods and advanced molecular diagnostics, as elaborated further in this review.

These new methods offer enhanced sensitivity compared with conventional techniques. They also provide improved specificity for accurate detection. These innovations enable earlier detection, improved genotyping, and more accurate outbreak tracking. By overcoming traditional serology and RT-PCR limitations, these molecular tools support timely public health responses and are gradually being integrated into surveillance systems. Although traditional diagnostics focus on single targets (N protein for measles IgM detection, N gene for measles RT-PCR amplification, E1 protein for rubella IgM, and E1 gene for rubella RT-PCR), newer methods demonstrate both target conservation and target expansion strategies as discussed below. Target conservation approaches maintain proven diagnostic targets while enhancing detection through improved assay chemistry and methodological innovations. Conversely, target expansion strategies broaden the scope of detection by incorporating multiple antigens or comprehensive genomic analysis.

## Chemiluminescence immunoassay

Chemiluminescence immunoassay (CLIA) is a highly sensitive and rapid diagnostic method using light-emitting chemical reactions to detect specific antibodies or antigens. This technology represents target conservation with methodological advancement. It utilizes the same N protein and E1 glycoprotein antigens as traditional ELISA but employs chemiluminescent detection for enhanced sensitivity and automation capabilities. It is especially useful for diagnosing measles and rubella by accurately identifying IgM antibodies, making it ideal for high-throughput and time-sensitive testing. Steve et al.^131^ compared CLIA and ELISA for detecting IgM antibodies against measles, mumps, rubella, CMV, EBV, and HHV-1/2 in 345 samples.^131^ CLIA was highly comparable to ELISA for measles (kappa = 0.86) and mumps (kappa = 0.92), with moderate agreement for rubella (0.52), CMV (0.57), EBV (0.50), and HHV-1/2 (0.47). After adjusting for prevalence bias, agreement improved for rubella (0.64), CMV (0.65), EBV (0.60), and HHV-1/2 (0.88). The study concluded that both assays can be used for IgM detection, with the choice depending on laboratory setup, throughput, and expertise. A meta-analysis found that DiaSorin LIAISON CLIA demonstrated the highest diagnostic accuracy for measles IgM detection, with 97% pooled sensitivity, 98% specificity, and a diagnostic odds ratio of 2,559.67.^131^ Most rubella IgM assays showed sensitivities and specificities above 90%. Tests like Euroimmun GP and DiaSorin had low heterogeneity. This indicates reliable performance across different platforms.

IgM-based assays, although highly sensitive and rapid, face inherent limitations related to antibody kinetics and cross-reactivity.^129^ The measles N protein shares structural homology with other paramyxoviruses. This potentially causes false-positive reactions in individuals with recent mumps or parainfluenza infections.^182^ Epitope mapping studies identified the most immunogenic regions of the N protein. These regions are located at amino acids 101–160 and 396–425. However, these sequences show 65%–70% identity with the mumps virus N protein, which may cause cross-reactivity.^115^^,^^130^^,^^131^^,^^167^^,^^183^

## Automatable focus reduction neutralization tests

The PRNT and ELISA are commonly used to assess measles immunity but differ in principle. ELISA measures antibody binding to viral components, whereas PRNT evaluates antibodies' ability to block viral infection. Although PRNT is the gold standard for detecting MeV-neutralizing antibodies, it is labor intensive, slow, and technically demanding.

To address these limitations, the focus reduction neutralization test (FRNT) was developed using an ICA to detect infected cells. Vaidya et al. (2014) developed an ICA applied in FRNT to assess immunity against measles, mumps, and rubella.^123^ The assay was evaluated using 23 measles, 6 mumps, and 6 rubella isolates, 3 vaccine strains, and 24 clinical samples. The ICA-based FRNT enabled virus detection within 2–3 days. The test produced visible blue foci for easy interpretation. This offers a rapid and reliable method for neutralization testing. The method requires low resources, making it accessible. It has strong potential for sero-epidemiological studies. It can also assess pre- and post-vaccine immunity effectively. FRNT offers faster results, improved specificity, and reduced sample volume and can be automated using 96-well plates.^127^^,^^134^ Terletskaia-Ladwig et al. (2011) validated FRNT as an alternative to PRNT for assessing measles immunity using 50 serum samples analyzed by PRNT, FRNT, and ELISA.^135^ ELISA failed to detect low levels of anti-measles antibodies. It identified only 19 positive samples compared with 38 by PRNT and 37 by FRNT. The two neutralization assays demonstrated perfect correlation and similar sensitivity. The study concluded that FRNT is a suitable alternative to PRNT for evaluating immunological responses and vaccination efficacy, especially useful for immunocompromised individuals. FRNT exemplifies target conservation through technical innovation. It maintains the same neutralizing antibody targets as traditional PRNT. The method introduces automated detection systems for better efficiency. FRNT also requires reduced sample volumes compared with the traditional methods. This combination improves the overall laboratory efficiency while preserving diagnostic accuracy. However, FRNT is not well suited for high-throughput testing platforms or population-level seroprevalence studies.^134^

## Lateral flow-based RDTs

Lateral flow-based RDTs are increasingly adopted for detecting infections by targeting specific antibodies or antigens, as with malaria, COVID-19, and HIV.^184^^,^^185^^,^^186^ These tests operate at room temperature without complex electrical equipment. They eliminate the need for reverse cold chain systems during specimen transport. The tests require minimal technical training to perform. This makes them ideal for low-resource settings. They are also perfect for remote environments with limited infrastructure.

Lateral flow immunoassays operate on capillary action and antigen-antibody binding kinetics.^187^ The test device consists of four main components working together. The sample pad allows for specimen application. The conjugate pad contains gold nanoparticle-labeled detection antibodies. The nitrocellulose membrane has immobilized capture antibodies that form test and control lines. An absorbent pad maintains the capillary flow throughout the process.^187^ When the sample is applied, target antigens form immunocomplexes with gold-conjugated antibodies. This complex migrates along the membrane and is captured at the test line by immobilized antibodies. This produces a visible colored band indicating a positive result.^188^ Lateral flow RDTs represent target conservation with simplified detection. It targets the same measles N protein-specific IgM antibodies as laboratory EIAs. The tests use recombinant N protein antigens immobilized on the test line. It enables rapid point-of-care testing in under 30 min. Results are interpreted visually without requiring instrumentation. This approach maintains diagnostic equivalence with reference methods while enabling decentralized testing. The assay design incorporates both capture and detection antibodies specific for human IgM heavy chains. This ensures class-specific antibody detection and minimizes cross-reactivity with other immunoglobulin classes.^150^ The multi-matrix compatibility stems from the assay’s ability to detect IgM antibodies at concentrations typically found in different specimen types. These tests can utilize serum and oral fluid samples for flexible specimen collection.^140^^,^^141^ The oral fluid collection device uses a specialized pad. This pad absorbs approximately 1 mL of oral fluid. The fluid is then extracted in a buffer solution. The buffer contains stabilizers and antimicrobial preservatives. These components maintain sample integrity during transport.^189^ Oral fluid collection offers particular advantages for pediatric populations and community screening programs, as it eliminates the need for trained phlebotomists and reduces patient discomfort.^190^ In serum, the RDT demonstrated 91% sensitivity (69/76) and 94% specificity (88/94), whereas in oral fluid, the sensitivity and specificity were 90% (63/70) and 96% (200/208), respectively.^191^ The analytical sensitivity of lateral flow RDTs is typically 2- to 5-fold lower than those of laboratory EIAs. This is due to the visual detection limit of gold nanoparticles. The tests require approximately 108–109 particles per test line for reliable visual interpretation. However, this sensitivity threshold aligns well with clinically relevant antibody concentrations during acute measles infection. The assay demonstrates robust performance across a wide range of environmental conditions. It maintains stability at temperatures up to 40°C. The test also works at relative humidity up to 85%. This makes it suitable for tropical and subtropical deployment. No cold chain requirements are needed for storage or transport.^192^ MeV RNA could be recovered and genotyped from IgM-positive RDTs stored dry for 5 weeks at 20°C–25°C.^141^ The ability to recover viral RNA from dried RDT devices represents a significant advancement. This enhances surveillance capability substantially. It enables both serological diagnosis and molecular characterization from a single specimen.^193^ The nitrocellulose membrane acts as a nucleic acid preservation matrix, with viral RNA remaining stable through desiccation and storage. RNA extraction protocols have been optimized for direct processing of the test strip material, typically yielding sufficient template for RT-PCR amplification and subsequent genotyping. This dual functionality supports enhanced measles surveillance by providing both rapid diagnosis and molecular epidemiological data for outbreak investigation and virus tracking.^194^

The prototype was redesigned for capillary blood use, adhering to ASSURED (Affordable, Sensitive, Specific, User-friendly, Rapid and robust, Equipment-free, and Deliverable to end-users) criteria, and manufactured commercially.^195^ The ASSURED criteria provide a framework for evaluating point-of-care diagnostics in resource-limited settings. The redesigned prototype achieves these benchmarks through several key features. It offers cost-effective manufacturing at less than US$2 per test. The device works with fingerstick capillary blood, eliminating venipuncture requirements. It can be stored at ambient temperature between 2°C and 30°C. Results are available in 15–20 min. Visual interpretation requires no instrumentation. The device incorporates internal quality controls and procedural controls to ensure test validity and reduce user errors.^196^ Clinical evaluation against Enzygnost anti-MeV IgM EIA using 125 sera from Brazil’s measles surveillance and dengue cases showed 95% sensitivity and 98% specificity. Visual interpretation showed excellent reliability with strong agreement (kappa >0.9) across three independent readers, confirming field suitability without instrumentation.^197^ The high inter-reader agreement reflects the binary nature of visual interpretation and optimized gold nanoparticle density for clear band visualization. Studies demonstrate that color intensity correlates with antibody concentration, but the test is designed for qualitative interpretation with a distinct visual threshold. Training materials include photographic reference standards showing positive, negative, and invalid results to standardize interpretation across different users and lighting conditions.^197^ A current limitation is the absence of a rubella RDT, as most GMRLN laboratories test measles IgM-negative samples for rubella IgM. A combined measles-rubella IgM RDT is under development.^193^ The development of a multiplex measles-rubella RDT faces technical challenges related to cross-reactivity and test line optimization. The combined assay requires separate capture zones for measles and rubella antigens while maintaining sensitivity for both targets. Preliminary designs may utilize differential positioning of test lines or dual-channel formats to enable simultaneous detection. This advancement would significantly streamline surveillance workflows. It benefits regions implementing measles-rubella elimination programs. The technology reduces the need for sequential testing. It also improves diagnostic efficiency for febrile rash illness investigations.^193^

## Reverse transcription droplet digital PCR

Reverse transcription droplet digital PCR (RT-ddPCR) represents an advanced molecular diagnostic technique that combines the precision of digital PCR (dPCR) technology with the capability to detect RNA targets through reverse transcription.^198^ RT-ddPCR is a specialized application of dPCR. It specifically addresses diagnostic needs for RNA viruses like measles and rubella. The method provides direct, absolute, and precise measurements of viral RNA. It works without the need for standard curves or reference controls.^146^ RT-ddPCR utilizes droplet-based partitioning technology. The sample first undergoes reverse transcription to convert viral RNA into cDNA. The sample is then partitioned into approximately 20,000 nanoliter-sized droplets using oil-water emulsion technology. Each droplet acts as an individual PCR microreactor, enabling the detection and quantification of target viral sequences with unprecedented accuracy and sensitivity.^199^ Droplets are analyzed for fluorescent signals, with each droplet classified as positive (containing target) or negative (no target). The concentration is calculated using Poisson statistics based on the ratio of positive to negative droplets. This approach eliminates the need for standard curves and provides absolute quantification with superior precision compared with traditional methods. Wu et al.^144^ (2024) conducted groundbreaking research on RT-ddPCR technology. They established it as a viable method for measles and rubella detection. The team developed and validated multiplexed assays for this purpose. The assay can detect viral RNA in both clinical and environmental samples.^144^ The study developed sophisticated RT-ddPCR assays targeting specific genomic regions of measles, mumps, and rubella viruses, with particular focus on the MeV matrix (M) gene, mumps virus large (L) gene, and rubella virus glycoprotein 1 (RUBVgp1) gene. The researchers designed innovative strain-differentiation assays for measles detection. They used identical forward and reverse primers but different probe sequences. This approach distinguishes between wild-type strains (genotypes D8 and B3) and Edmonston vaccine strains. The distinction is made through amplitude-based discrimination. The measles wild-type assays (WT1 and WT2) were specifically designed to match circulating genotypes perfectly. They incorporate 2–3 mismatches to vaccine strains. This design enables clear separation of positive droplet clusters at different amplitude levels. The study achieved impressive analytical performance results. The limit of quantification was 590 copies/mL for measles and 460 copies/mL for rubella in wastewater matrices. The assays were successfully validated using wastewater samples. These samples were collected from manholes outside facilities experiencing active measles outbreaks. Importantly, the research demonstrated the practical utility of RT-ddPCR by confirming wild-type measles genotype D8 through both RT-ddPCR amplitude discrimination and subsequent Sanger sequencing validation. This established a robust workflow for both detection and molecular epidemiological characterization.

The South African National Institute for Communicable Diseases established a national wastewater surveillance network utilizing RT-ddPCR technology, processing over 3,350 samples.^147^ The study revealed that RT-ddPCR wastewater surveillance consistently detected viral circulation. This occurred in districts where traditional clinical surveillance systems failed to identify cases. Wastewater results showed positive detection in 26% of time-district pairs for hepatitis A. Detection rates were 8% for hepatitis E, 3% for measles, and 1% for rubella. These positive results occurred when clinical cases were reported in the same time frame. More significantly, the technology detected viral presence in wastewater when no clinical cases were reported. This occurred in 4% of time-district pairs for hepatitis A, 25% for hepatitis E, 3% for measles, and 4% for rubella. This demonstrates the superior sensitivity of environmental surveillance compared with passive clinical reporting systems. The network achieved consistent laboratory performance with specific turnaround times. Quantification results were available in 5–7 days. Sequencing confirmation took 3–4 weeks to complete. The laboratory processed 25–58 samples weekly. Rigorous quality control standards were maintained throughout the surveillance period.

RT-ddPCR offers transformative advantages for measles and rubella diagnostics through its unique combination of absolute quantification capabilities and superior analytical performance.^200^ The technology provides direct quantification of viral RNA without requiring standard curves, calibrators, or reference controls. This eliminates the variability and complexity associated with relative quantification methods. The method delivers exceptional sensitivity capable of detecting viral concentrations as low as 260–590 copies/mL.^144^ The droplet partitioning strategy not only concentrates target molecules to improve detection of low-abundance viral RNA typical in early infections but also provides enhanced resistance to PCR inhibitors commonly found in clinical and environmental specimens.^201^ Perhaps most significantly, RT-ddPCR enables unprecedented strain differentiation capabilities through amplitude-based discrimination. This allows real-time distinction between wild-type and vaccine measles strains. This represents a critical capability for accurate case classification in elimination programs. It also enables differentiation between natural infections and vaccine reactions. The technology supports robust multiplexing for simultaneous detection of multiple viral targets in a single reaction, increasing diagnostic efficiency while reducing sample volume requirements and costs.^202^^,^^203^ Additionally, RT-ddPCR demonstrates exceptional reproducibility with lower inter- and intra-assay variability compared with qPCR, making it highly suitable for standardized protocols across different laboratory settings and supporting quality assurance programs in global surveillance networks.

Despite its remarkable analytical capabilities, RT-ddPCR faces several significant limitations that currently restrict its widespread adoption for routine measles and rubella diagnostics.^204^^,^^205^ The most critical barrier is the complete absence of commercial RT-ddPCR kits specifically designed and validated for measles and rubella detection, forcing laboratories to develop custom assays using laboratory-developed protocols that require extensive in-house validation and platform-specific optimization.^206^ The technology demands substantial capital investment with equipment costs ranging from US$60,000–200,000. Per-test costs are US$35–60, significantly higher than those of established molecular diagnostic methods. The technology also requires specialized training in droplet generation, data interpretation, and quality control procedures specific to dPCR technology.^207^ RT-ddPCR exhibits reduced throughput capacity compared with high-throughput qPCR systems. It is due to the time-intensive droplet generation process and limited automation capabilities. This makes it less suitable for large-scale surveillance programs or high-volume clinical laboratories.^208^ The technology also suffers from a narrower dynamic range due to the finite number of droplets generated per reaction, typically around 20,000. This potentially requires sample dilution for high viral load specimens. The method faces constraints with sample processing, including sensitivity to RNA quality. It is unsuitable for large amplicons and has potential issues with highly degraded viral material.^209^ Furthermore, the current lack of comprehensive clinical validation studies, WHO approval for routine surveillance applications, and standardized protocols across laboratories limit the technology’s integration into established diagnostic workflows and public health surveillance systems.^102^^,^^210^

## Isothermal amplification and CRISPR-Cas-based approach

Reverse transcription loop-mediated isothermal amplification (RT-LAMP) operates on the principle of strand displacement DNA synthesis using *Bst* DNA polymerase. This enzyme possesses both 5′ to 3′ polymerase activity and strand displacement activity but lacks 5′ to 3′ exonuclease activity. This enables continuous amplification at constant temperature through the formation of stem-loop structures with multiple loops, creating a characteristic cauliflower-like structure of concatenated inverted repeats of the target sequence.^211^ The assay targets nucleotides 400–800 of the measles N gene using six primers. These primers recognize eight distinct sequences within this region. This provides enhanced specificity compared with conventional RT-PCR. The six-primer system includes two outer primers (F3/B3), two inner primers (FIP/BIP), and two loop primers (LF/LB). Together, they recognize eight distinct sequences. The outer primers F3 and B3 initiate the reaction by binding to the 5′ and 3′ ends of the target sequence, whereas the inner primers FIP and BIP contain complementary sequences to both the sense and antisense strands, creating the initial stem-loop structures. The loop primers LF and LB accelerate the reaction by binding to the loop portions of the stem-loop DNA structures. This reduces the amplification time from 60–90 min to 40 min^212^ This design creates multiple loop structures during amplification, generating pyrophosphate by-products detectable by turbidity or lateral flow devices without requiring fluorescent probes.^213^ The accumulation of pyrophosphate ions during DNA synthesis reacts with magnesium ions to form magnesium pyrophosphate precipitate. This increases turbidity that can be monitored in real-time using turbidimeters or observed visually. Additionally, pH indicators such as phenol red can be incorporated to provide colorimetric detection, changing from pink (alkaline) to yellow (acidic) as protons are released during DNA synthesis.^214^ A rapid and sensitive RT-LAMP assay coupled with a lateral flow device was developed for MeV detection.^136^ The assay developed by Xu et al. (2016) operates at 58°C for 40 min, with results visualized on a disposable strip. It demonstrated a detection limit of 8.8 copies/μL, comparable to real-time RT-PCR. Validation using 494 clinical samples showed high concordance with RT-PCR results. The method is simple, cost-effective, and suitable for routine surveillance in CDC laboratories.^136^ RT-LAMP’s multi-primer design, while providing robustness, also increases the risk of primer-dimer formation and non-specific amplification. The method’s tolerance for sequence mismatches (up to two nucleotides per primer) can lead to cross-amplification of closely related sequences or detection of vaccine strains when wild-type-specific assays are intended. To further enhance specificity and reduce false-positive results inherent in isothermal amplification methods, CRISPR-Cas systems have been integrated as secondary confirmation steps, providing programmable nuclease-based target validation. A study by Pinchon et al. (2024) combines reverse transcription recombinase polymerase amplification (RT-RPA) with CRISPR-Cas12a-based detection and a lateral flow device. The reaction completes in under 1 h at 42°C.^148^ The system employs guide RNAs (gRNAs) designed against conserved regions of the measles N gene, enabling single-nucleotide discrimination for variant detection and vaccine strain differentiation. The Cas12a nuclease requires a protospacer adjacent motif (PAM) sequence (5′-TTTV-3′ where V = A, G, or C) flanking the target site. Upon recognition of the target sequence by the crRNA (CRISPR RNA), the Cas12a ribonucleoprotein complex undergoes conformational changes that activate its *trans*-cleavage activity. This activation enables the nuclease to cleave single-stranded DNA reporters non-specifically, including fluorescent or biotin-labeled probes used in lateral flow detection. The *trans*-cleavage activity is strictly dependent on target recognition, ensuring high specificity and minimal background signal.^215^ gRNAs are designed against conserved 20-nucleotide sequences within the measles N gene that contain appropriate PAM sites, typically targeting nucleotides 800–1,200 to avoid vaccine strain cross-reactivity. It demonstrated a detection limit of 31 measles RNA copies per reaction. Diagnostic evaluation using 27 RT-PCR-positive and 29 negative saliva samples showed 96% sensitivity and 100% specificity, with an overall accuracy of 98%. Compared with conventional RT-PCR requiring thermal cycling equipment, these isothermal methods offer a promising point-of-care diagnostic tool for MeV, especially in low-resource settings. RT-LAMP can be performed using simple heating blocks or even body heat incubation, while the CRISPR-Cas12a system provides single-nucleotide discrimination capability superior to mismatch-tolerant isothermal amplification methods. The integration of lateral flow detection eliminates the need for gel electrophoresis or fluorescent detection systems, making these approaches particularly suitable for resource-limited settings and point-of-care applications.^216^ However, CRISPR-Cas12a detection requires precise gRNA design and is sensitive to SNPs within the target sequence. The system’s dependence on PAM sequences limits target selection, and emerging viral variants may escape detection if mutations occur within the 20-nucleotide gRNA binding site or adjacent PAM sequence. Both RT-LAMP and CRISPR-Cas12a exemplify target conservation with enhanced specificity, maintaining the proven N gene target for measles while introducing improved recognition mechanisms that offer superior discrimination between wild-type and vaccine strains compared with conventional RT-PCR.

## Multiplex bead assay

The multiplex bead assay (MBA) operates on the principle of suspension-based immunoassays. It uses color-coded polystyrene microspheres that are 5.6 μm in diameter as a solid-phase support. Each distinct bead population is internally dyed with unique ratios of two fluorescent dyes. The beads are conjugated with specific viral antigens, such as measles or rubella proteins.^217^ The assay workflow involves incubating patient serum with antigen-coupled microsphere mixtures to allow antibody-antigen binding. This is followed by detection using biotinylated secondary antibodies and streptavidin-phycoerythrin conjugates that create fluorescent signals proportional to antibody concentration.^218^ A dual-laser flow cytometer system simultaneously identifies microsphere populations through internal dye classification (635 nm laser) and quantifies reporter signal intensity to determine antibody concentrations (532 nm laser). This enables measurement of up to 100 different analytes in a single reaction using minimal serum volume (1–5 μL).^142^ It offers significant advantages over traditional ELISA, including high-throughput capability and the ability to analyze up to 500 antigens in a single assay, depending on the instrument.^219^

For measles and rubella surveillance, the MBA has been developed and validated using recombinant measles nucleoprotein and whole-virus lysates. The assay incorporates multiple measles antigens, including full-length N protein, truncated H protein fragments, and F protein domains, whereas rubella detection utilizes recombinant E1 glycoprotein and whole virus lysates to capture the complete antibody response spectrum. The assay provides high-quality seroprevalence data and shows strong correlation with ELISA and PRNT, supporting its use in large-scale seroprotection studies. The MBA represents a target expansion approach, moving beyond single-antigen detection to comprehensive automated immune profiling. This platform incorporates multiple measles antigens (N, H, F proteins) and rubella antigens (E1, capsid proteins) simultaneously. It provides high-throughput multiplexing capabilities and quantitative antibody titer determination. The system offers cost efficiency by replacing multiple individual assays. It also reduces inter-assay variability through automated processing. This makes it particularly valuable for comprehensive immune status assessment and outbreak investigations where multiple pathogen screening is required from limited sample volumes when compared with the traditional single-target ELISA methods. However, MBA has limitations, including the need for specialized equipment, trained personnel, and advanced data analysis tools. Additionally, cross-reactivity, antigen standardization, and accessibility in low-resource settings may affect broader implementation.

## Microfluidic chip-based detection

Microfluidic chip-based detection for measles and rubella represents an emerging and highly promising diagnostic approach that leverages microfabrication technology to create miniaturized, integrated platforms capable of performing complex biochemical analyses within confined microscale channels.^220^^,^^221^ The technology operates on the principle of precise fluid manipulation and control within microchannels (typically 1–1,000 μm in dimensions). This enables complete sample-to-answer analysis in a single, portable device that significantly reduces sample consumption, shortens reaction time, and improves detection sensitivity compared with conventional laboratory-based methods.^222^ Recent developments have demonstrated two primary microfluidic approaches for measles and rubella detection. The first approach uses digital microfluidic (DMF) systems that employ electrostatic forces to manipulate discrete droplets for automated immunoassays. The second approach utilizes continuous-flow microfluidic chips that integrate multiple analytical steps. These chips include nucleic acid amplification and antibody detection capabilities.^223^^,^^224^

A groundbreaking DMF platform, DMF-ELISA, developed for field deployment, has achieved measles and rubella IgM/IgG detection directly from whole blood samples. Knipes et al. (2022) reported field validation results of DMF-ELISA technology tested in Kenya (2016) and the Democratic Republic of Congo (2017). It demonstrated IgG and IgM detection performance with 81%–88% concordance compared with reference laboratory assays.^139^ The turnaround time for complete analysis was only 35 min using a portable “MR Box” system weighing just 4 kg. Complementary advances by Huang et al. (2021) include two-stage isothermal amplification microfluidic chips that combine RPA with LAMP for parallel detection of viral RNA, achieving limits of detection as low as 10 copies with 100% specificity and sensitivity for MeV using minimal sample volumes (2.1 μL for RPA and 10.6 μL for LAMP reactions).^143^ Although RPA alone can yield false-positives due to primer-dimer formation, and LAMP has lower sensitivity, the two-stage method improves sensitivity by 10- to 100-fold over LAMP alone. This study presents the first combination of RPA and LAMP to harness RPA’s high sensitivity and LAMP’s high specificity, overcoming their individual limitations.

This microfluidic platform demonstrates hybrid target utilization, maintaining conventional gene targets while integrating multiple amplification and detection technologies. This shows how advanced platforms achieve innovation through methodological integration rather than target modification.^225^ In addition, it offers transformative advantages, including point-of-care deployment capability and reduced infrastructure requirements. The system provides rapid turnaround times suitable for outbreak response. It has cost-effective mass production potential and can perform multiplexed detection of multiple pathogens simultaneously. This makes the technology particularly valuable for resource-limited settings and emergency response scenarios where traditional laboratory infrastructure is unavailable.^226^ However, microfluidic chip-based detection systems face significant limitations, including a lack of commercial availability, with no US Food and Drug Administration-approved or WHO-validated systems currently available.^220^^,^^227^ The technology shows reduced sensitivity (81%–88%) compared with reference methods, potentially missing outbreak cases.^143^ Complex manufacturing requires specialized facilities and high costs. Implementation needs specialized training, and limited technical support exists in remote settings.^223^ The systems show sensitivity to environmental conditions affecting device performance. They have limited dynamic range and multiplexing capabilities compared with laboratory platforms. Per-test costs are higher than those of established methods. Challenges exist with reagent stability in miniaturized formats and potential cross-contamination issues. The technology depends on reliable power sources and faces difficulties with waste disposal and maintenance in resource-limited environments.

## Next-generation sequencing

Whole-genome sequencing (WGS) represents the most comprehensive molecular approach for measles and rubella surveillance, utilizing NGS technologies to capture and analyze complete viral genomes (15.9 kb for measles, 9.8 kb for rubella) rather than traditional single-gene targets.^12^^,^^228^ With the dramatic reduction in genetic diversity of circulating MeV strains and the predominance of only two genotypes (B3 and D8) globally, standard N-450 nucleotide sequencing can no longer provide adequate resolution for outbreak investigation and transmission tracking.^229^ The WHO-GMRLN now recommends WGS approaches as essential tools for countries approaching or maintaining elimination status, particularly when traditional molecular epidemiology cannot demonstrate the absence of endemic transmission.^228^ The technique employs three primary methodologies. The first is amplicon-based approaches using overlapping PCR products spanning the entire genome. The second is probe enrichment systems utilizing biotinylated oligonucleotides for targeted viral sequence capture. The third is direct shotgun metagenomic sequencing from clinical specimens.^230^ Probe enrichment, the most efficient approach, uses 120 overlapping probes for measles and 80 probes for rubella. This achieves >1,000-fold viral sequence enrichment compared with shotgun sequencing through hybridization at 65°C. This is followed by streptavidin bead capture and host nucleic acid removal. WGS of the MeV enables precise genotyping, transmission tracking, and outbreak investigations. Probe enrichment panels target all six viral genes (N, P, M, F, H, L) simultaneously, with 120 overlapping probes covering the complete 15.9-kb genome. For rubella, 80 probes span the 9.8-kb genome, focusing on structural genes including E1, E2, and capsid regions. Traditional platforms are limited by cost, infrastructure, and turnaround time. Oxford Nanopore Technologies (ONT) offers promising alternatives for decentralized surveillance in resource-limited settings. Real-time WGS analysis during active outbreaks has been demonstrated through mobile laboratory deployment, enabling immediate phylogenetic analysis and transmission mapping to guide public health interventions. Portable ONT MinION devices allow sequencing directly at outbreak sites, with results available within 6–24 h.^231^ NGS represents the most comprehensive target expansion approach, moving beyond single-gene targets (N gene for measles, E1 for rubella) to complete viral genome characterization. This whole-genome strategy enables simultaneous analysis of all six MeV genes (N, P, M, F, H, L) and all rubella virus genes. This provides unprecedented genetic resolution for outbreak investigation, transmission tracking, and variant detection that single-gene approaches cannot achieve. Zubach et al. (2025) developed WGS protocols for MeV genotype D8 using ONT with 1-kb amplicons, comparing Guppy/Dorado basecallers with Q-score thresholds of 20/25. Complete genomes were recovered at 10 copies/μL, although consensus errors emerged at 100 copies/μL.^149^ Among 32 clinical samples, GQ20 yielded the highest completeness (75%), sequence identity (84.4%), and reproducibility (97.4%) with <1 nucleotide error per genome. Contemporary WGS protocols have been validated across multiple circulating genotypes. The success rates vary by viral load: >90% genome coverage achieved for 79.5% of specimens using combined NGS and Sanger approaches, whereas NGS-only success drops to 71.2% due to difficulties with non-coding regions, particularly the matrix/fusion non-coding regions (M/F-NCR).^149^^,^^232^ Limitations include consensus errors at lower viral loads and evaluation limited to genotype D8. Schulz et al. (2022) compared three NGS approaches: PCR amplicon enrichment, shotgun metagenomics, and probe enrichment. MeV-specific probe enrichment showed the highest efficiency with complete genome coverage in ∼70% of specimens and minimal off-target reads. This approach eliminates labor-intensive amplicon generation and applies across circulating genotypes. Cost analysis estimated US$75 per sample, with enrichment contributing US$7.^145^ Bucris et al. (2021) performed WGS directly from clinical specimens using cost-efficient sample allocation based on viral load. High-resolution surveillance across >4,000 cases in Israel used 41 high-MeV-content urine samples (Ct ≤ 20), demonstrating feasibility without culture.^233^ Cost-optimized sequencing strategies allocate sequence reads based on viral load: ∼1 million reads for high-viral-content samples (Ct < 18) to achieve complete genome coverage at 10× depth. This reduces per-sample costs while maintaining analytical quality.^234^^,^^235^ The GMRLN has established standardized WGS protocols with quality metrics requiring minimum 90% genome coverage and depth thresholds for reliable variant calling.^236^ Proficiency testing programs now include WGS components, with 762 laboratories in the global network building capacity for extended sequencing.^107^ NGS-based MeV WGS limitations include dependence on high-quality specimens, poor Ct-sequencing success correlation, GC-rich region bias, high costs, and the need for probe updates for emerging genotypes.^237^ WGS has proven critical for distinguishing continuous transmission from repeated importations, with applications during major outbreaks including >4,000 cases in Israel demonstrating the feasibility of large-scale genomic surveillance. Integration with epidemiological data through tools like named strains and distinct sequence identifiers enhances transmission chain analysis.^232^^,^^238^

Gene target selection significantly impacts assay performance. N gene-based assays for measles offer high sensitivity but may show reduced specificity in recently vaccinated individuals due to vaccine strain cross-reactivity.^174^ H gene targets provide better genotype discrimination but require higher viral loads for reliable detection.^132^ For rubella, E1 gene assays balance sensitivity and specificity, although 5′ UTR targets may be preferred for specimens with low viral loads typical of rubella infections.^115^^,^^177^

Current diagnostic limitations highlight the need for rapid, sensitive tools for early infectious periods, vaccinated individuals, and low-incidence regions.^132^ Cloud-based analysis pipelines and automated genotyping tools facilitate deployment in resource-limited settings. Major development challenges include insufficient funding and limited private sector incentives.^239^

## Ensuring quality in molecular diagnostics: The role of mEQA

As molecular diagnostics become integral to MeV and RuV detection, genotyping, and surveillance, ensuring accuracy is critical. The mEQA program, launched by the US CDC in 2014, supports this within the WHO-GMRLN.^240^ With over 100 participating laboratories worldwide, the mEQA assesses performance across nucleic acid-based platforms and protocols. Its phased implementation with progressively stringent criteria enables laboratories to maintain high-quality molecular testing for case confirmation and viral surveillance.^107^ The program informs capacity-building strategies, including targeted training, equitable reagent access, and standardized data submission to MeaNS for measles and RubeNS for rubella.^241^^,^^242^

The mEQA program faces several challenges. RNA copy numbers in proficiency panels do not fully represent clinical specimen ranges.^243^ MeV panels contain normal-to-high RNA, whereas RuV panels have very high copy numbers. This limits sensitivity assessment, which will be addressed in 2024 with unscored samples containing lower RNA copy numbers.^240^ The 3-month turnaround time for reporting conflicts with the 2-month timeline for sequence data submission to the database, prompting separate reports for detection and sequencing results in 2025. Additional challenges include panel stability during shipping, language barriers, staff turnover, equipment maintenance difficulties, and potential result sharing between laboratories. The reduction in circulating MeV genotypes has diminished the routine genotyping data value, prompting analysis of larger sequence regions like M/F-NCR or whole genomes.^244^ Although Sanger sequencing remains standard for routine genotyping and M/F-NCR sequencing, increased use of NGS platforms like Illumina and ONTs necessitates separate EQA programs for these newer technologies.^230^^,^^245^ Laboratory’s flexibility in assay choice, although beneficial, complicates mEQA administration as laboratories resist adopting new reference genes or sequencing platforms. The COVID-19 pandemic has accelerated the shift from Sanger to newer sequencing platforms, requiring further adaptation of mEQA evaluation and scoring systems. The mEQA remains foundational for reliable molecular surveillance underpinning global measles and rubella elimination strategies.

## Implementation challenges and limitations

Based on the comprehensive review of diagnostic advances for measles and rubella, several overarching limitations constrain the translation of innovative technologies into routine clinical practice and global surveillance systems. The most significant barrier is the substantial gap between technological capabilities and real-world implementation, particularly in resource-limited settings where the burden of disease remains highest. Advanced molecular platforms such as CRISPR-Cas12a systems, dPCR, and NGS demonstrate exceptional analytical performance. However, they require specialized infrastructure, skilled personnel, and substantial capital investment. These requirements exceed the capacity of most endemic countries.^246^ Cost-effectiveness remains a critical limitation for emerging technologies. Per-test expenses are often 5 to 10 times higher than conventional methods. The economic benefits of enhanced sensitivity and specificity have not been adequately quantified in elimination settings. Regulatory approval pathways for novel diagnostic platforms are fragmented and lengthy. Most innovative assays remain in research phases without WHO validation. They also lack integration into established quality assurance frameworks.^98^ The persistent lack of commercial availability for promising technologies, exemplified by the absence of validated RT-ddPCR kits or standardized CRISPR-based assays, reflects insufficient market incentives for diagnostic development in vaccine-preventable diseases. Technical limitations include reduced to moderate sensitivity of point-of-care platforms compared with laboratory-based methods, contamination risks in isothermal amplification techniques, and the complexity of implementing comprehensive genomic surveillance in countries with limited bioinformatics capacity.^247^

## Future perspectives

The advancement of hybrid diagnostic approaches represents a promising direction. These approaches combine the accessibility of rapid tests with the analytical power of molecular methods. Investment in comprehensive health system strengthening initiatives is essential. These initiatives must address the underlying infrastructure deficits limiting diagnostic implementation. This includes power supply reliability, cold chain maintenance, and workforce development programs. The establishment of tiered diagnostic networks could optimize resource utilization and diagnostic coverage. These networks would strategically deploy advanced technologies at regional reference laboratories. They would also support simplified testing at peripheral sites. Integration of AI and machine learning approaches for automated result interpretation, quality control, and epidemiological analysis offers potential solutions to the technical expertise limitations currently constraining implementation.^248^

Future research priorities should focus on developing cost-effective manufacturing processes for innovative diagnostics that support technology adoption in low-income settings.^249^^,^^250^ Studies should conduct large-scale operational research to demonstrate real-world effectiveness. Although the diagnostic technologies reviewed in this article demonstrate significant technical advances, their application to personalized therapies for measles and rubella remains largely theoretical. Key limitations include the lack of validated therapeutic targets and insufficient clinical validation of diagnostic technologies. These technologies have not been specifically validated for therapeutic decision making. Cost-effectiveness concerns and regulatory barriers exist that have not been pursued for measles and rubella. Future research should focus on developing validated therapeutic targets. Studies should demonstrate the utility of advanced diagnostics in personalizing measles and rubella treatment approaches. The integration of these technologies with personalized medicine remains a promising but unproven concept. This concept requires substantial further investigation.^251^

Also, the future of measles and rubella diagnostics is intrinsically linked to vaccination program optimization through real-time diagnostic feedback loops. The WHO’s IA2030 framework and the Foundation for Innovative New Diagnostics specifically call for diagnostic-vaccination integration through three operational modalities. These are pre-campaign immunity assessment, real-time outbreak response, and post-campaign effectiveness evaluation.^72^ The Malaysia field trial (2019–2020) demonstrated that diagnostic-vaccination integration reduced public health response time by 5 days.^141^ Future platforms will build on this model by deploying integrated mobile units combining rapid diagnostic testing with immediate vaccination capabilities. Healthcare workers achieved 99% inter-reader agreement for RDT interpretation, maintaining 80% knowledge retention 9 months post-training, establishing the feasibility of field deployment. Drawing from Zimbabwe's 2010 outbreak response, researchers evaluated 103 suspected cases using RDTs and successfully performed viral genotyping directly from the test strips. This approach demonstrates how integrated diagnostic systems can provide real-time strain identification to inform vaccine strain selection and enhance contact tracing efforts.^3^ This approach eliminates the current 3- to 7-day delay between case detection and strain identification that limits targeted response effectiveness. Extending Knipes et al.'s Democratic Republic of Congo field validation showing 81%–88% concordance with reference methods, future implementation will deploy portable “MR Box” systems at mobile vaccination units to achieve 35-min sample-to-answer testing capabilities. These integrated platforms will incorporate GPS tracking for real-time immunity mapping during vaccination campaigns, enabling immediate vaccination decision making for tested individuals and creating dynamic population immunity profiles to guide targeted intervention strategies.^139^ The Brazil surveillance evaluation demonstrated 95% sensitivity and 98% specificity for RDT testing using sera from 97 suspected cases, establishing the accuracy threshold needed for pre-vaccination serological screening.^197^ Future campaigns will deploy rapid serological testing to map population immunity before SIAs, optimizing vaccine allocation based on actual immunity gaps rather than estimated coverage data. Current network standards require 97% of laboratories to achieve passing scores in annual proficiency testing.^107^ Future integration will mandate that point-of-care platforms achieve ≥95% agreement with reference laboratory methods, while integration protocols must maintain current surveillance sensitivity while reducing response times. Quality assurance programs will be extended to field-deployed diagnostic-vaccination teams to ensure consistent performance standards across all testing environments, from central laboratories to community-level deployment sites. Future targets should align with the WHO’s 24-h confirmation guidelines and 2030 elimination objectives.^47^

Ultimately, achieving the full potential of diagnostic innovations for global measles and rubella elimination will require coordinated international efforts. These efforts must address not only technological development but also the multifaceted systemic barriers. These barriers prevent equitable access to life-saving diagnostic tools across diverse healthcare environments. These barriers include inadequate healthcare infrastructure in resource-limited settings and insufficient funding mechanisms for procurement and deployment. Gaps exist in healthcare worker training and capacity building. Regulatory hurdles delay approval and implementation, while supply chain challenges limit sustained availability. Success will depend on collaborative partnerships between international organizations, governments, manufacturers, and research institutions. These partnerships must create comprehensive solutions that ensure innovative diagnostic technologies can reach the most vulnerable populations. This is critical where measles and rubella remain endemic threats.

## Conclusion

Measles and rubella remain significant public health challenges despite decades of elimination efforts. Although immunization has substantially reduced disease burden, ongoing outbreaks highlight persistent gaps in surveillance systems and diagnostic capabilities, especially in low-resource settings. The importance of immunization in terms of health and economy, along with several programs including mEQA and IA2030, was highlighted as key initiatives supporting global elimination strategies. This review comprehensively examined the evolution of diagnostic approaches for measles and rubella, from conventional methods like serology and RT-PCR to emerging technologies. Among rapidly growing nucleic acid-based technologies, isothermal amplification and CRISPR-Cas platforms show particular promise for field-deployable diagnostics, offering rapid, highly specific detection capabilities that could transform point-of-care testing. The demonstrated benefits of these emerging technologies, including enhanced specificity, improved accessibility, reduced costs, and operational simplicity, provide compelling evidence for their integration into global surveillance systems. Political commitment, decentralization, and simplification of diagnostic platforms, including the development of innovative nucleic acid-based approaches, coupled with investment in quality-assured, scalable, and accessible solutions, are essential for minimizing surveillance gaps. Equipping global health systems with next-generation diagnostic tools and robust quality control frameworks will significantly improve outbreak response capabilities, guide more effective immunization strategies, and accelerate progress toward measles and rubella elimination compared with reliance on conventional methods alone.

## Data availability

This article does not contain any datasets generated during the current study. All data discussed in this review are derived from previously published sources, which are cited throughout the manuscript.

## Acknowledgments

We acknowledge the World Health Organisation for funding the project (202814880-1/Unit Reference IVD/CDS/SEARO). We acknowledge Dr. Sudhir Khanal from WHO-SEARO, New Delhi, for valuable inputs in the manuscript. Dr. Paul Rota from the 10.13039/100000030US CDC is acknowledged for information and inputs on the measles and rubella viruses. The author Shivani Sharma acknowledges the fellowship support from the Department of Biotechnology, Ministry of Science and Technology, India. We would also like to acknowledge the 10.13039/100018337South Asian University for providing the institutional support.

## Author contributions

The authors, S.S. and Y.R.P., planned and drafted the manuscript.

## Declaration of interests

No conflict of interest to declare.
